# Silencing of Claudin-11 Is Associated with Increased Invasiveness of Gastric Cancer Cells

**DOI:** 10.1371/journal.pone.0008002

**Published:** 2009-11-24

**Authors:** Rachana Agarwal, Yuriko Mori, Yulan Cheng, Zhe Jin, Alexandru V. Olaru, James P. Hamilton, Stefan David, Florin M. Selaru, Jian Yang, John M. Abraham, Elizabeth Montgomery, Patrice J. Morin, Stephen J. Meltzer

**Affiliations:** 1 Department of Medicine, The Johns Hopkins University School of Medicine, Baltimore, Maryland, United States of America; 2 Department of Pathology, The Johns Hopkins University School of Medicine, Baltimore, Maryland, United States of America; 3 Laboratory of Cellular and Molecular Biology, National Institute on Aging, Baltimore, Maryland, United States of America; George Washington University School of Medicine, United States of America

## Abstract

**Background:**

Claudins are membrane proteins that play critical roles in tight junction (TJ) formation and function. Members of the claudin gene family have been demonstrated to be aberrantly regulated, and to participate in the pathogenesis of various human cancers. In the present study, we report that claudin-11 (*CLDN11*) is silenced in gastric cancer *via* hypermethylation of its promoter region.

**Methodology/Principal Findings:**

Levels of *CLDN11* methylation and mRNA expression were measured in primary gastric cancer tissues, noncancerous gastric mucosae, and cell lines of gastric origin using quantitative methylation-specific PCR (qMSP) and quantitative reverse transcriptase-PCR (qRT-PCR), respectively. Analyses of paired gastric cancers and adjacent normal gastric tissues revealed hypermethylation of the *CLDN11* promoter region in gastric cancers, and this hypermethylation was significantly correlated with downregulation of *CLDN11* expression *vs.* normal tissues. The *CLDN11* promoter region was also hypermethylated in all gastric cancer cell lines tested relative to immortalized normal gastric epithelial cells. Moreover, *CLDN11* mRNA expression was inversely correlated with its methylation level. Treatment of *CLDN11*-nonexpressing gastric cancer cells with 5-aza-2′-deoxycytidine restored *CLDN11* expression. Moreover, siRNA-mediated knockdown of *CLDN11* expression in normal gastric epithelial cells increased their motility and invasiveness.

**Conclusions/Significance:**

These data suggest that hypermethylation of *CLDN11*, leading to downregulated expression, contributes to gastric carcinogenesis by increasing cellular motility and invasiveness. A further understanding of the mechanisms underlying the role of claudin proteins in gastric carcinogenesis will likely help in the identification of novel approaches for diagnosis and therapy of gastric cancer.

## Introduction

Gastric cancer (GC) remains the second most-common cause of cancer deaths globally. It is one of the most lethal malignancies and a leading cause of cancer deaths in developing countries, with overall 5-year survival rates below 20% [1], [2].

Studies of GCs and their preneoplastic precursor lesions have identified several genetic and epigenetic alterations, including microsatellite instability, point mutations, and loss of heterozygosity (LOH) affecting tumor suppressor genes (TSGs) [3]–[6]. Nevertheless, the molecular pathogenesis of GC is still incompletely understood.

Epigenetic alterations are extremely important in cancer development and progression [7]. Transcriptional inactivation of tumor suppressor genes via aberrant promoter hypermethylation of CpG islands, causing permanent gene silencing, is a major epigenetic mechanism of TSG inactivation.

Previously, studies have been published on promoter hypermethylation in GCs and their premalignant precursors [8]–[10]. p16INK4A and p15INK4B were among the first genes to show hypermethylation in GCs [11]. Our group and others subsequently discovered hypermethylation of the hMLH1 DNA mismatch repair gene in GCs exhibiting frequent microsatellite instability (MSI-H) [12]–[14]. We also showed that hypermethylation of the E-cadherin (CDH1) gene occurs frequently in GCs [15].

A pilot microarray-based genome-wide search conducted by our group, performed to discover novel epigenetically silenced genes in gastric carcinogenesis, identified claudin-11 (*CLDN11*), a tight junction (TJ) protein, as a potential target of epigenetic inactivation in gastric cancers (unpublished data).

Claudin-11 belongs to the family of claudin proteins, which contains more than 23 members. Members of the claudin family are expressed in a highly tissue-specific manner in a variety of normal and neoplastic tissues [16]. Claudins are transmembrane proteins that play crucial roles in TJ formation and function. TJs are intercellular junctions critical in the paracellular transport of solutes, as well as in maintaining cell polarity. Tumor cells commonly exhibit structural and functional deficiencies in their TJs [17].

In recent years, a number of studies have demonstrated aberrant expression of claudin proteins in various cancer types [18], [16]. Several of these studies found dysregulation of claudin protein expression *via* promoter hypermethylation. Hypermethylation-induced silencing of claudin-7 expression was previously reported in breast [19] and colorectal [20] carcinomas. Claudin-6 is also epigenetically silenced in breast cancer [21], while claudins -3 and -4 are epigenetically regulated in ovarian cancer cells [22], [23].

The current study identifies and reports, to our knowledge for the first time, that the *CLDN11* promoter region is hypermethylated in GC tissues and cell lines. Moreover, while *CLDN11* mRNA was expressed in all primary noncancerous gastric mucosal tissues, as well as in an immortalized normal gastric epithelial cell line, it was silenced in all GC tissues and cell lines examined. Interestingly, siRNA-mediated downregulation of *CLDN11* in *CLDN11*-expressing gastric cells was also associated with cancer-related phenotypic changes, specifically increased cell motility and invasiveness.

## Materials and Methods

### Cell Lines and Clinical Tissue Specimens

Immortalized human normal gastric epithelial cells (HFE145) were obtained from Dr. Duane T. Smoot (Howard University) and GC cell lines AGS, SIIA, MKN28, KATOIII, and SNU-1 were obtained from ATCC. All cell lines were cultured and maintained in RPMI 1640 medium supplemented with 10% fetal bovine serum and 1% antibiotic-antimycotic solution (Invitrogen).

Paired primary gastric normal and tumor tissues were collected at the Johns Hopkins Hospital (JHH). Specimens were snap-frozen immediately after resection.

### Ethics Statement

Johns Hopkins University (JHU) Institutional Review Board (IRB) approval was obtained for all the cases included in the study. Cases were obtained from JHU surgical pathology under JHU IRB-approved exemption 02-07-19-05e under rule 45 CFR 46.101(b), which waives the requirements for obtaining patient consent.

### Purification and Preparation of Genomic DNA and Total RNA

Genomic DNA was extracted from snap-frozen tissue samples or cell lines using a DNeasy Blood & Tissue Kit (QIAGEN, Valencia, CA) according to the manufacturer's protocol. Total RNA was extracted with Trizol reagent (Invitrogen). Extracted DNA and RNA were quantified using a NanoDrop ND-1000 Spectrophotometer (NanoDrop, Wilmington, DE).

### Quantitative Methylation-Specific PCR (qMSP) for Claudin-11

Genomic DNAs obtained from 36 patient samples comprising 18 GC and 18 matched noncancerous stomach mucosal (NS) tissues, as well as from various gastric cell lines, were subjected to qMSP. qMSP was performed as described previously, with minor modifications [24]. In brief, bisulfite treatment of genomic DNAs was performed using an EpiTect Bisulfite Treatment Kit (QIAGEN, Valencia, CA). An MSP amplicon and TaqMan probe to detect completely methylated DNA were designed to include multiple CpG sites in the 5′-UTR region of *CLDN11* gene. *CLDN11*-specific primers and probe sequences used were: forward primer 5′ CGCGATTGGTCGGCGCGTTTC 3′; reverse primer 5′ GACGAAAACAACAACGCTACT 3′; TaqMan probe 5′TCGGAGTCGCGGGGTTTAAAGAG 3′. CpGenome Universal Methylated DNA (Chemicon International, Temecula, CA) served as a positive control, and serial dilutions of it were used to plot a standard curve. qMSP with TaqMan probe were performed on an iQ5 thermal cycler (BioRad, Hercules, CA) using iQ Supermix (*ibid.*). Fifty cycles of PCR amplification starting with 50 ng of bisulfite-treated genomic DNA were performed in triplicate, according to the manufacturer's protocol. Duplex PCR with β-actin (ACTB) primer and probe sequences containing no CpGs were performed for normalization. The primer and probe sequences used were as published previously [24]. Normalized methylation value (NMV) was defined as follows: NMV = (*CLDN11-S*/*CLDN11-FM*)/(*ACTB-S*/*ACTB-FM*) * 100, where *CLDN11-S* and *CLDN11-FM* represent *CLDN11* methylation levels in the sample and fully methylated DNAs, respectively, while *ACTB-S* and *ACTB-FM* correspond to *β-actin* in the sample and fully methylated DNAs, respectively. Whole-genome amplified DNA (WGA) was used as an unmethylated negative control.

### Quantitative Reverse Transcription-PCR Analysis

The *CLDN11* RT-PCR amplicon was designed to overlap an intron-exon boundary in order to exclude genomic DNA (*g*DNA) amplification. Primer sequences were as published previously [18]. One-step qRT-PCR was performed as described previously [24], using a Quantitect SYBRA RT-PCR kit (QIAGEN, Valencia, CA), according to the manufacturer's protocol. *CLDN11*expression was normalized to *β* -actin expression. Total RNA from HFE145 cells was used for the standard curve. (*CLDN11-S*/*CLDN11-C*)/(*ACTB-S*/*ACTB-C*), where *CLDN11-S* and *CLDN11-C* represent *CLDN11* mRNA expression levels in the test sample and control mRNAs, respectively, while *ACTB-S* and *ACTB-C* correspond to *β-actin* expression levels in the test sample and control mRNAs, respectively.

### 5-Aza-2′-Deoxycytidine (5-Aza-dC) Treatment

AGS is a GC cell line manifesting hypermethylation of the *CLDN11* promoter in conjunction with absent *CLDN11* mRNA expression. 5-aza-2′-deoxycitidine (5-aza-dC) treatment of cells was performed as described previously [24]. In brief, the GC cell line AGS (ATCC catalog number CRL-1739) was seeded at a density of 2×10^5^ cells/ml in a T-75 flask. Twenty-four hours later, cells were treated with 1 µM 5-aza-dC for 72 hours. Media containing 5-aza-dC was replaced with freshly prepared media every 24 hours. Cells were then harvested for total RNA extraction. Total RNAs from AGS cells before and after 5-aza-dC treatment were subjected to quantitative real-time RT-PCR analysis.

### Small Interfering RNA Knockdown Experiments

*CLDN11*-specific siRNA oligos were purchased from Ambion, Inc. (Austin, TX). The HFE145 cell line, which is *CLDN11* positive, was selected to study the effect of claudin-11 knockdown on migration and invasion properties of gastric cells. Experiments were conducted as described previously (Agarwal *et al.*, 2005). Cells cultured in 6-well plates were transfected with siRNA duplexes using LipofectAMINE 2000 (Invitrogen), following the manufacturer's instructions. Mock-transfections and nonspecific siRNA duplexes were used as negative controls. Cells were treated for 48 to 72 hours to allow maximum knockdown, after which they were either harvested for Western blot analysis or used for migration and invasion assays.

### Western Blot Analysis of Claudin-11 in Gastric Cell Lines

Confluent cell cultures were washed with HBSS (Invitrogen) and whole cell lysates were made using lysis buffer: 62.5 mmol/L Tris-HCl (pH 6.8), 10% glycerol, and 2% SDS. Protein concentration was determined using a bicinchoninic acid (BCA) assay kit (Pierce, Rockford, IL). Twenty micrograms of total proteins were separated by 10% to 20% SDS-PAGE on Tris-Glycine gels (Invitrogen) and transferred to polyvinylidene difluoride membranes (Millipore Corp., Bedford, MA). The membranes were blocked with 5% nonfat dry milk, washed in TBST buffer, and probed with an anti-claudin-11 antibody (Zymed, San Francisco, CA). Blots were then washed and incubated in horseradish peroxidase-conjugated secondary antibody (anti-rabbit IgG: 1∶10,000; Amersham Pharmacia Biotech, Piscataway, NJ). For detection, chemiluminescence was carried out using an enhanced chemiluminescence kit (Amersham Pharmacia Biotech).

### Cell Invasion and Migration Assay

Invasiveness of siRNA-transfected cells was determined using Matrigel-coated invasion chamber inserts (24-well-format with 8-µm pores, BD Biosciences) using a modified Boyden chamber assay [25]. Cells were cultured to approximately 80% confluency and serum-starved overnight. On the day of the assay, cells were trypsinized and viable cell counts taken. Approximately 50,000 cells were plated onto the top of each of each filter in serum-free medium. An equal volume of the same medium containing 20% FCS was placed in the lower chamber (*i.e.*, the well beneath the filter) to act as a chemoattractant. Assay plates were incubated at 37°C for up to 48 hrs. Cells that did not migrate or invade through the pores of the Transwell inserts were manually removed with a cotton swab. Cells present at the bottom of the membrane were fixed in cold methanol for 10 min and then stained with 0.01% crystal violet in 20% ethanol. After 10 min incubation, the filters were washed thoroughly in water and suspended in 200 µL of 5% acetic acid and 5% methanol. Colorimetric readings were taken at OD_595_. Experiments were repeated at least three times, with triplicates in each experiment. To assess cell migration, assays were carried out essentially as above, except that cells were plated on top of uncoated (Matrigel-free) inserts.

### Statistical Analysis

Statistical analyses were performed using Student's t-test (SPSS, version 16), with *p*<0.05 considered statistically significant.

## Results and Discussion

**Figure 1:**
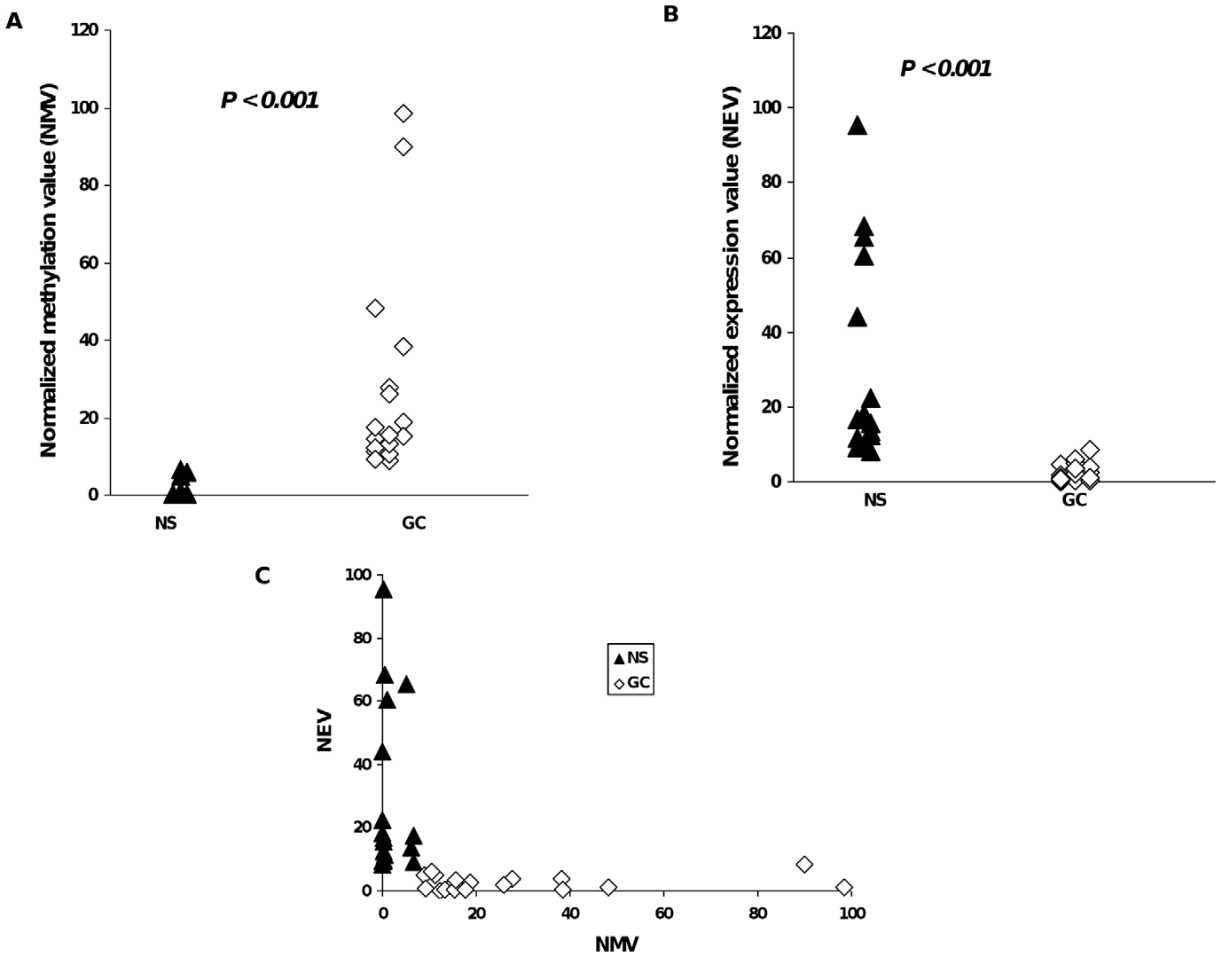
Promoter methylation and mRNA expression levels of claudin-11 (*CLDN11)* in clinical specimens. This figure illustrates the promoter methylation and mRNA expression levels of *CLDN11* in paired gastric cancer (GC) and noncancerous gastric mucosal (NS) tissues. A) Quantitative methylation-specific PCR (qMSP) for *CLDN11* in primary tissues. Genomic DNA extracted from 18 GC and 18 matched NS tissues were subjected to qMSP analysis using MSP amplicon and TaqMan probe designed to include multiple CpG sites in the 5′-UTR region of *CLDN11* gene. CpGenome Universal Methylated DNA (Chemicon International, Temecula, CA) was used as a fully methylated positive control. Duplex PCR with β-actin (ACTB) primer and probe sequences containing no CpGs were performed for normalization. Normalized methylation value (NMV) was defined as follows: NMV = (*CLDN11-S*/*CLDN11-FM*)/(*ACTB-S*/*ACTB-FM*) * 100, where *CLDN11-S* and *CLDN11-FM* represent *CLDN11* methylation levels in the sample and fully methylated DNAs, respectively, while *ACTB-S* and *ACTB-FM* correspond to *β-actin* in the sample and fully methylated DNAs, respectively. This one-dimensional scatterplot demonstrates significantly high *CLDN11* promoter methylation levels in the GC specimens when compared to the NS specimens (*P*<*0.001*). Whole-genome amplified DNA (WGA) used as an unmethylated negative control did not show any amplification. The *P* value was calculated using the paired Student's t-test. B) *CLDN11* mRNA expression in gastric tissues. Total RNA extracted from 18 paired NS and GC specimens were subjected to *CLDN11* specific RT-PCR. *CLDN11* mRNA expression was normalized to *β* -actin mRNA expression in each sample. The *P* value was calculated using the paired Student's t-test. This plot demonstrates that the *CLDN11* mRNA expression levels were significantly lower to non-detectable in the GC specimens, while most of the corresponding NS specimens had detectable *CLDN11* mRNA levels (*P*<*0.001*). C) 2D-scatter plot of promoter methylation and mRNA expression values in GC tissues. *CLDN11* mRNA expression silencing is associated with promoter hypermethylation in GC patients. This two-dimensional scatterplot demonstrates NEV values (Y-axis) and NMV values (X-axis) in the 18 paired NS and GC specimens.

We first tested our hypothesis that the *CLDN11* promoter is hypermethylated and that this hypermethylation correlates inversely with *CLDN11* mRNA expression in primary GC tissues. Paired primary gastric normal and tumor tissues were collected at the Johns Hopkins Hospital (JHH). Specimens were obtained immediately after resection and snap-frozen until use. Only cases obtained with informed consent as approved by the Institutional Review Board (IRB) were included in this project. Genomic DNAs were obtained from 36 clinical specimens, comprising 18 GC and 18 matched noncancerous gastric mucosal (NS) tissues. *CLDN11* promoter methylation levels in these samples were analyzed using quantitative real-time methylation-specific PCR (qMSP). **Figure 1A** displays the normalized methylation value (NMV), *i.e.*, the ratio of methylated value of the *CLDN11* promoter region in each specimen to a fully methylated control DNA. *CLDN11* NM*Vs* were significantly higher in GC specimens than in their matching NS tissues (*P*<0.001). To assess whether *CLDN11* promoter hypermethylation in GCs was associated with silencing of *CLDN11* expression, *CLDN11* mRNA levels were measured using quantitative real-time (qRT–PCR) in RNAs extracted from the 36 specimens used for methylation analysis. As demonstrated in **Figure 1B**, normalized expression values (NEVs) of *CLDN11* in GC specimens were significantly lower than in paired NS samples (*P*<0.001). This data establishes that *CLDN11* promoter hypermethylation correlates with diminished or silenced *CLDN11* mRNA expression in primary GCs.

**Figure 2:**
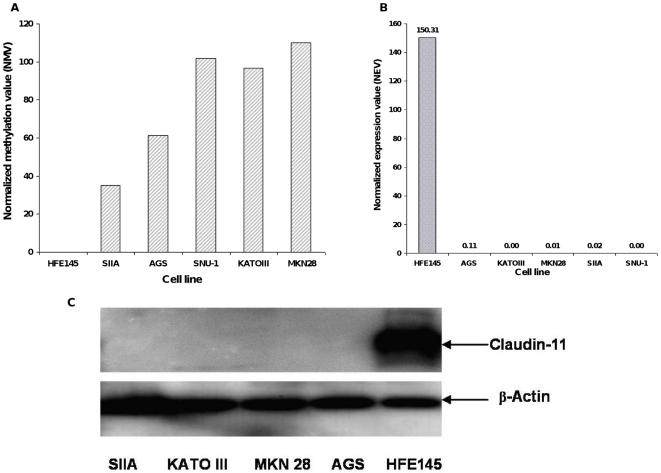
Analysis of promoter methylation, mRNA and protein expression of claudin-11 in gastric cell lines. This figure illustrates the claudin-11 promoter methylation, mRNA expression levels and protein expression in gastric cells lines. A) Quantitative methylation-specific PCR (qMSP) for *CLDN11*. Genomic DNAs isolated from immortalized human normal gastric epithelial cells (HFE145) and GC cell lines AGS, SIIA, MKN28, KATOIII, and SNU-1 obtained from ATCC were analyzed by qMSP. This Figure illustrates that the promoter region of *CLDN11* gene is hypermethylated in all GC cell lines relative to HFE145 cells. B) *CLDN11* mRNA expression in gastric cell lines. Total RNAs from different gastric cell lines were subjected to quantitative real-time RT-PCR analysis. As can be seen in this figure, HFE145 cells expressed very high levels of *CLDN11* mRNA, while all five cancer cell lines tested had very low or undetectable *CLDN11* mRNA expression. C) Western blot analysis of claudin-11 expression in gastric cell lines. Total cell lysates obtained from various gastric cell lines were probed with the anti-claudin-11 antibody. This figure illustrates that while the immortalized normal gastric epithelial cell line, HFE145, expressed abundant claudin-11 protein, it could not be detected in various GC cell lines. Anti-β-actin antibody was used as a loading control.

Next, we evaluated *CLDN11* promoter methylation and expression in gastric cell lines. Immortalized human normal gastric epithelial cells (HFE145) and GC cell lines AGS, SIIA, MKN28, KATOIII, and SNU-1 were studied. All GC cell lines tested (AGS, SIIA, MKN28, KATOIII, and SNU-1) demonstrated promoter hypermethylation, whereas no methylation was observed in immortalized normal HFE145 cells **(** **Figure 2A** **)**. *CLDN11* mRNA levels were assessed using qRT-PCR on RNAs purified from the cell lines, while claudin-11 protein expression was analyzed by Western blotting. As shown in **Figure 2B**, all 5 cancer lines exhibited no detectable expression of *CLDN11* mRNA or protein, while HFE145 cells manifested high *CLDN11* expression levels. This finding establishes that *CLDN11* is coordinately hypermethylated and downregulated in GC cell lines relative to normal gastric epithelial cells (NGECs).

**Figure 3:**
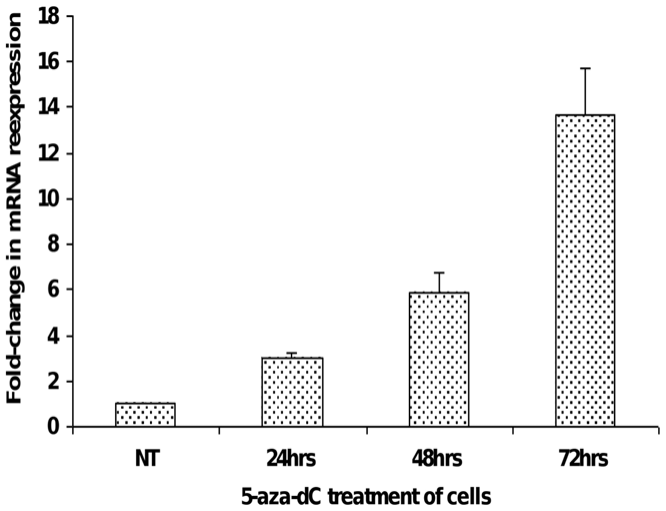
*CLDN11* mRNA re-expression in AGS GC cells after 5-aza-2′-deoxycytidine (5-aza-dC) treatment. AGS is a GC cell line manifesting hypermethylation of the *CLDN11* promoter in conjunction with absent *CLDN11* mRNA expression. These cells were treated with 5-aza-dC, a global demethylating agent. Total RNA from AGS cells before and after 5-aza-dC treatment were subjected to quantitative real-time RT-PCR analysis for *CLDN11* mRNA expression. The *Y-axis* represents the average fold change in expression levels of *CLDN11* mRNA after 5-aza-dC treatment at various time points, when compared with the untreated cells. AGS cells, when treated with 5-aza-dC, exhibited a time-dependent increase in *CLDN11* mRNA expression up to 72 hrs of treatment. This restoration of *CLDN11* expression after 5-aza-dC treatment supports our hypothesis that claudin-11 is silenced by promoter hypermethylation in GC cells.

To further validate silencing of *CLDN11* expression by hypermethylation, we treated the GC cell line AGS with the demethylating agent, 5-aza-2-deoxycytidine (5-aza-dC, 1 µM), for varying time intervals. Total RNAs extracted before *vs.* after treatment were subjected to qRT-PCR for *CLDN11*. As can be seen in **Figure 3**, at each time point, *CLDN11* mRNA became re-expressed upon treatment with 5-aza-dC, confirming that *CLDN11* is silenced by promoter hypermethylation in AGS GC cells.

**Figure 4:**
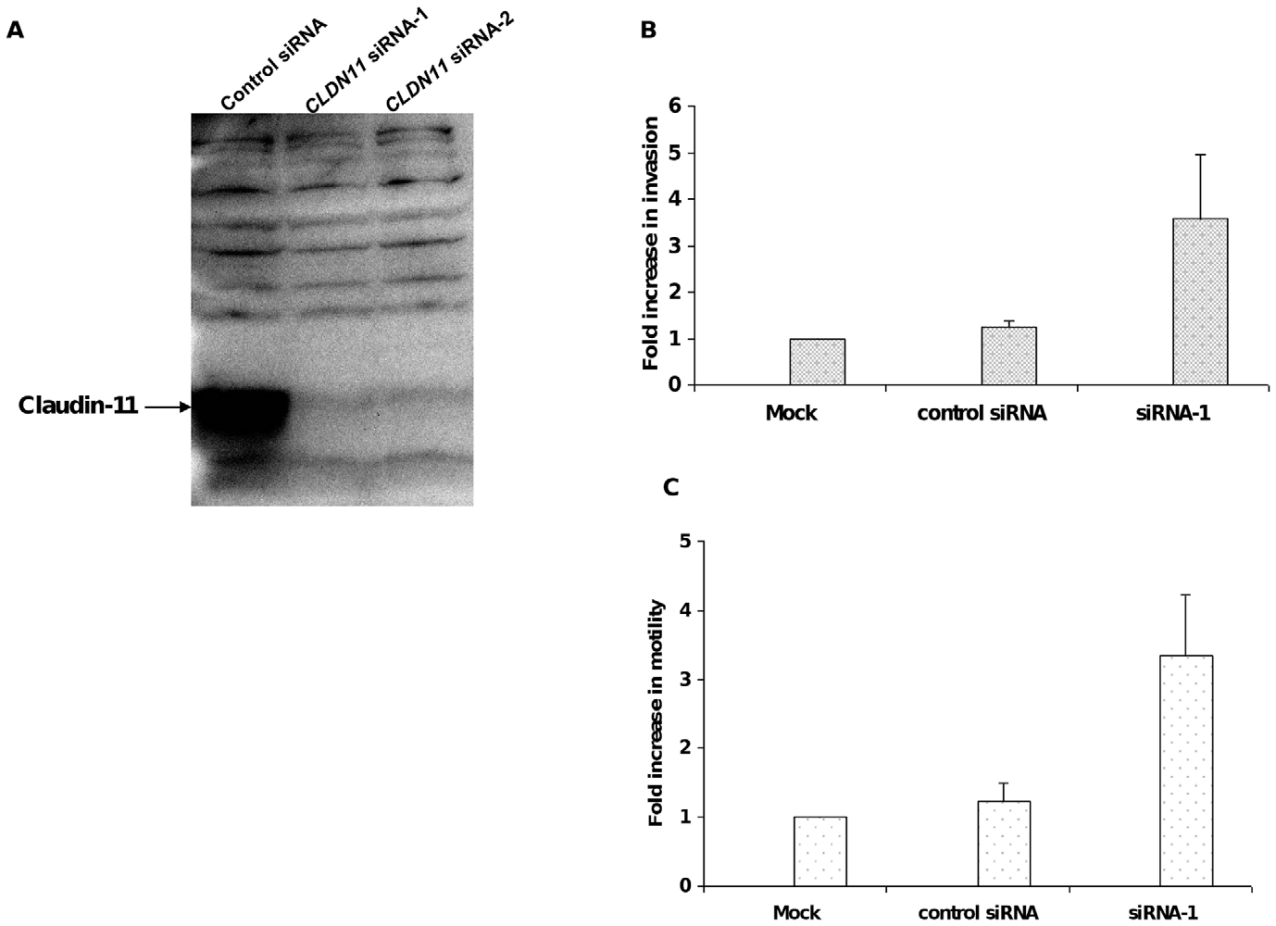
Small interfering RNA knockdown experiments. HFE145 cell line, which is claudin-11 positive was selected to study the role of claudin-11 knockdown on migration and invasion properties of gastric cells. Cells were transfected with *CLDN11* specific siRNA oligos. Mock transfections and nonspecific siRNA duplexes were used as the negative controls. A) Western blot analysis of siRNA mediated claudin-11 knock-down in HFE145 cells. HFE145 cells were transfected with *CLDN11*- specific and non-specific control siRNA duplexes, as described in Materials and Methods. After 48 to 72 hours, total cell lysates were prepared and analyzed for claudin-11 expression. Transfection of claudin-specific siRNA oligos resulted in>90% reduction in expression of the protein, whereas the levels of claudin protein in the control cells were not significantly altered. B) Boyden chamber cell invasion assay. The invasiveness of the siRNA-transfected cells were determined using matrigel coated invasion chamber, using a modified Boyden chamber assay. Experiments were repeated at least three times, with triplicates in each experiment. The data represented here is the average fold change in invasion of the siRNA-transfected cells when compared with the mock transfections. As is demonstrated in this figure, siRNA knockdown of claudin-11 leads to an increase in cell invasion of HFE145 cells. C) Two-chamber cell migration assay. The effects of claudin-11 knockdown on migration of the HFE145 cells were compared by measuring the number of cells migrating through the uncoated filters (instead of matrigel-coated filters). The bars in this figure represent mean fold change in migration of siRNA-transfected cells compared with the mock-transfected control cells. As can be seen in this figure, a reduction in claudin-11 protein levels is associated with increased motility of the cells.

Members of the claudin protein family have been implicated in the regulation of cell adhesion, invasion and migration of cancer cells [25]–[27]. Therefore, we next investigated whether *CLDN11* influences cell motility or invasiveness. HFE145 cells, which express abundant *CLDN11*, were chosen to study effects of *CLDN11* knockdown on the invasive and migratory properties of gastric epithelial cells. Transient siRNA transfections were carried out using *CLDN11-*specific siRNA duplexes. Transfection with *CLDN11*-specific siRNA duplexes efficiently repressed claudin-11 protein levels by>90%, whereas expression remained unchanged in mock- or control siRNA-treated cells (**Figure 4A**). Cell motility and invasion assays were conducted on siRNA-transfected cells using a modified Boyden-chamber assay system [25]. Interestingly, as shown in **Figures 4B and 4C**, inhibition of *CLDN11* expression in HFE145 cells significantly increased the migratory and invasive potentials of these cells, respectively.

The current study thus confirms the hypothesis that *CLDN11*, a tight junction protein, is silenced in GC via promoter hypermethylation, and these data support the involvement of *CLDN11* in the control of GC cell invasion and migration.

Promoter hypermethylation is known to be associated with transcriptional silencing of certain genes. DNA methylation can interfere with binding of transcription factors whose binding sites contain CpG dinucleotides. Using the TFSEARCH software program, we scanned the *CLDN11* sequence found to be hypermethylated in gastric cancer tissues and cell lines, *i.e*., the qMSP amplicon (-104 to +4 bases relative to the transcriptional start site for *CLDN11*). This scan identified putative binding sites for Sp1 and GATA-1 and GATA-2. Previous studies have shown that hypermethylation of promoter DNA contributes to silencing of *CLDN3* and *CLDN4* in ovarian cell lines, in part *via* methylation-induced disruption of binding of Sp1 to its cognate binding site (22, 23). In addition, members of the GATA family have been shown to be positive regulators of *CLDN11* transcription [28]. Further studies are now warranted to evaluate whether hypermethylation of these sites interferes with the binding of transcription factors, and how such disruption may influence *CLDN11* gene transcription.

Tumor cells typically exhibit structural and functional deficiencies in their TJs [17]. These deficiencies are associated with a loss of polarity and differentiation. Another important link between TJs and cancer is a loss of TJ integrity, with consequent leakage or transport of pro-tumorigenic substances (such as growth factors or nutrients) into developing tumor cell primordia, promoting tumor growth [29]. In addition, the loss of polarity, differentiation, and adhesive properties associated with impaired TJ function in cancer may be critical in acquiring a metastatic phenotype [30]. Studies have suggested that anomalies in TJ-associated proteins may represent epithelial-mesenchymal transition (EMT), thereby changing the invasiveness and motility of cancer cells.

Modulations in expression of TJ-associated proteins, particularly claudin proteins, have been shown in a number of cancers. Recent studies by us and others have shown alterations in claudin protein regulation in various epithelial cancers [18]. Members of the claudin gene family are expressed in a highly tissue-specific, as well as a very developmental stage-specific, manner. In addition, depending on cancer type, the expression of claudin proteins can be either upregulated or downregulated in cancer cells. For example, several claudin proteins are upregulated in colon, ovarian, pancreatic and prostate cancers, while some are downregulated in breast cancer and head and neck cancers [16], [18]

Studies have previously reported changes in expression profiles of members of the claudin family to be associated with GC. Serial analysis of gene expression (SAGE) identified the *CLDN18* gene as downregulated in GCs possessing an intestinal phenotype [31]. The *CLDN23* gene is also downregulated in intestinal-type GCs [32]. Conversely, Cunningham *et al.* reported *CLDN4* expression to be increased in intestinal metaplasia and gastric epithelial dysplasia, identifying this gene as a marker of GC precursor lesions [33]. In the current study, we found *CLDN11* expression to be downregulated or silenced in GC *via* hypermethylation of its promoter region. Moreover, we found that unlike *CLDN18* and *CLDN23*, *CLDN11* expression was downregulated in GC patient specimens as well as in cell lines, irrespective of diffuse *vs.* intestinal subtype.

Claudin-11, also known as oligodendrocyte-specific protein, was first identified to be specifically expressed in the tight junction strands of oligodendrocytes in brain and in Sertoli cells of rats and mice [34], [35]. Loss of claudin-11 expression, leading to disruption of the TJ barrier, is associated with neurological and reproductive deficits [36]. A recent study reported overexpression and mislocalization of *CLDN11* from the blood–testis barrier in Sertoli cells to be associated with testicular intraepithelial neoplasia in men [37]. Data in the current study establishes that *CLDN11* is expressed in normal gastric tissues and immortalized NGECs. However, its complete functions in normal stomach as well as in gastric carcinogenesis remain unclear.

In an attempt to explore the possible involvement of *CLDN11* in GC, we studied phenotypic changes associated with silencing of *CLDN11* expression in *CLDN11-*expressing gastric epithelial cells (HFE145). Very interestingly, siRNA-mediated knockdown of *CLDN11* resulted in increased cell motility and invasion.

Previous studies have reported cancer-specific phenotypic changes to be associated with modulations in claudin expression in various cancer types. Overexpression of claudin-3 and claudin-4 proteins in ovarian cancer cell lines results in increased invasion by these cells [25]. In colon cancer, increased expression of claudin-1 has been reported, and changes in claudin-1 expression exert significant effects on the growth of xenografted tumors and metastases in athymic nude mice [27]. On the other hand, claudin-7 downregulation in breast cancer has been associated with increased cellular discohesion and the ability of breast cancer cells to disseminate [19]. In addition, experiments in pancreatic cell lines showed that expression of claudin-4 leads to reduced invasiveness, tumorigenicity, and metastatic potential of these cells [38]. The reasons for these discrepancies in effect are presently unclear, but may be related to tissue-specific differences in claudin function or even to variations in response of different cell lines. Clearly, members of the claudin family are known to be expressed in a tissue-specific manner and may exercise divergent effects.

Although in recent years it has become clear that TJs and TJ-associated proteins have extensive and diverse functional and physiological activities in normal and cancerous conditions, molecular mechanisms underlying these activities remain unclear. TJs are sophisticated intercellular apparatuses capable of recruiting signaling proteins, thereby regulating various cellular processes including cell growth, differentiation, and tumorigenesis [39], [40]. Claudin proteins directly associate with discrete signal transduction pathways by interacting with signaling molecules, such as atypical protein kinase C and Rho proteins, as well as with other PDZ domain-containing proteins. In ovarian cancer, claudins modify tumor invasion by regulating MMP activity [25].

In summary, data from the current study identify *CLDN11* as a target of epigenetic modification, as well as a promising biomarker for gastric cancer early detection, diagnosis, and therapy. These findings also suggest involvement of *CLDN11* downregulation in carcinogenesis *via* promotion of cell invasion and motility. The mechanisms for this phenomenon are subject to further investigation.
